# Real-World Visual and Refractive Results of Two Different Presbyopia Correcting Intraocular Lenses

**DOI:** 10.3390/jcm14228259

**Published:** 2025-11-20

**Authors:** Sarah Hinterberger, Cornelia Artmayr, Karanpreet Multani, Kamran M. Riaz, Seth M. Pantanelli, Klemens P. Kaiser, Achim Langenbucher, Matthias Bolz, Jascha A. Wendelstein

**Affiliations:** 1 Medical Faculty, Johannes Kepler University Linz, Altenberger Strasse 69, 4040 Linz, Austria; 2 Department for Ophthalmology and Optometry, Kepler University Hospital GmbH, Krankenhausstrasse 9, 4020 Linz, Austria; 3 Dean McGee Eye Institute, University of Oklahoma, Oklahoma City, OK 73019, USA; 4 Department of Ophthalmology, Penn State College of Medicine, Hershey, Derry, PA 17033, USA; 5 Department of Ophthalmology, Goethe-University Frankfurt, 60590 Frankfurt, Germany; 6 Institute of Experimental Ophthalmology, Saarland University, Kirrberger Str. 100/22, 66424 Homburg, Germany; 7 Department of Ophthalmology, Ludwig-Maximilians-Universität, 80336 Munich, Germany; 8 Institut für Refraktive und Ophthalmo-Chirurgie (IROC), 8002 Zurich, Switzerland

**Keywords:** cataract surgery, biometry, presbyopia-correcting IOL, refractive outcomes

## Abstract

**Background/Objectives**: To investigate visual acuity, refractive outcomes and the predictive accuracy of modern intraocular lens (IOL) power calculation formulas in eyes implanted with two presbyopia-correcting IOLs: trifocal Zeiss AT LISA TRI and the nondiffractive EDOF Teleon Comfort. **Methods**: This retrospective consecutive chart review included 115 patients who underwent uncomplicated bilateral cataract surgery and received either the LISA TRI (*n* = 56) or Comfort (*n* = 59). Biometric measurements were obtained preoperatively, and refractive outcomes were assessed 1, 3, and 6 months postoperatively. Postoperative spherical equivalent (SEQ) was compared to predicted SEQ using the ESCRS calculator and IOLCON platform. Outcome measures included mean prediction error and mean absolute error (MAE). Distance-corrected visual acuity (VA), uncorrected VA, defocus curves, preferred viewing distances, contrast sensitivity, and photopic reading speed were also analyzed. **Results**: All formulas performed better in the LISA TRI group, with significantly lower MAE and higher proportions of eyes within ±0.50 diopters (D). Systematic prediction error offsets were observed for three formulas (K6, Castrop, Hoffer QST) in the LISA TRI group and for all five formulas in the MF15 group. Refractive stability was achieved by 3 months for the LISA TRI, while 20% of Comfort eyes continued to show SEQ shifts > 0.50 D at 3 months. Defocus equivalent (DEQ) had lower proportions of eyes within ±0.50 D than SEQ. **Conclusions**: The LISA TRI demonstrated superior predictive accuracy, faster refractive stabilization, and stronger near performance than the Comfort. These findings support the importance of IOL-specific constant optimization and highlight the need for incorporating DEQ into routine refractive outcome evaluation.

## 1. Introduction

Presbyopia represents the most common refractive disorder in individuals over 40 years of age, and its correction remains an important focus in modern refractive cataract surgery [1,2]. Multifocal intraocular lenses (IOL), including trifocal IOLs, have been designed to provide satisfactory uncorrected visual acuity across (UCVA) various distances and provide maximal depth of field (DOF), but their success critically depends on precise IOL power calculations due to their sensitivity to even minor residual refractive errors [1]. However, in optics, there is no gain without a price—the maximal DOF achieved with multifocal IOLs (mIOLs) comes at the expense of reduced contrast sensitivity and photic phenomena, particularly glare and halos [3,4,5]. When photic phenomena raise concerns in a particular patient, extended depth-of-field (EDOF) IOLs offer extended DOF (compared to monofocal IOLs) with less photic phenomena (compared to mIOLs) but at the cost of reduced near vision, often necessitating reading glasses [1]. In our approach, we recommend trifocal IOLs for patients prioritizing maximum DOF without hesitation regarding photic disturbances, while EDOF IOLs are preferred for those seeking an extended DOF but who are apprehensive about halos and willing to accept the need for near-vision correction.

Multiple systematic reviews and meta-analyses, including those by the Cochrane Eyes and Vision Group and recent network meta-analyses, have demonstrated that both trifocal and EDOF IOLs achieve excellent uncorrected distance visual acuity (UDVA), with trifocal IOLs generally providing superior uncorrected near visual acuity (UNVA) and EDOF IOLs offering advantages at intermediate distances [3,5,6,7,8,9,10,11,12]. The American Academy of Ophthalmology has noted that both lens types improve spectacle independence compared to monofocal IOLs, though trifocal IOLs may yield higher rates of spectacle independence for near tasks, while EDOF IOLs may be associated with fewer photic phenomena such as halos and glare [6,13]. Patient-reported outcomes, including quality of life and satisfaction, are generally high for both lens types, with differences in subjective visual disturbances and spectacle dependence influencing individual patient preferences [3,6,10,13].

In this context, our study focuses on the refractive results of two presbyopia correcting IOLs with distinct optical designs. The Lentis Comfort LS-313 MF15 (including its stigmatic and astigmatic versions, LS-313-MF15 T0–T6, Teleon GmbH, Spankeren, The Netherlands) is a rotationally asymmetric refractive EDOF IOL that offers reliable far and intermediate visual performance [14]. Conversely, the stigmatic and astigmatic Zeiss AT LISA TRI 839/939 (Carl Zeiss Meditec AG, Jena, Germany) is an mIOL engineered to optimize vision across multiple focal points [15].

The present study aims to assess the visual and refractive outcomes and evaluate various IOL power calculation formulas’ performance for these two presbyopia correcting IOLs under routine clinical conditions.

## 2. Patients and Methods

### Study Design

This retrospective study was conducted in accordance with the local ethics committee (Ethics Committee of the Medical Faculty, Johannes Kepler University Linz, approval number, EK-Nr: 1258/2025; 29 August 2025) and the ethical principles outlined in the Declaration of Helsinki, as revised in 2013. The requirement for individual informed consent was waived by the Committee due to the retrospective nature of the study and use of de-identified data.

All data were anonymized at the source (removing names and birthdates) and compiled in Microsoft Excel (v.16.12, Microsoft Corporation, Redmond, WA, USA). The consecutive case series included all patients who underwent uncomplicated bilateral cataract surgery between November 2018 and May 2021 using either presbyopia correcting IOL at the study institution, with a postoperative refraction performed one month (21–45 days), 3 months (60–120 days), and 6 months (150–210 days) after the second eye surgery. No patients who received these IOLs and had all measurements performed over the study period were excluded from the analysis. The two presbyopia correcting IOL models used were the Zeiss AT LISA TRI 839/939 and the Teleon Comfort LS-313-MF15/LS-313-MF15 T0–T6.

Both eyes were targeted for emmetropia using the closest negative predicted postoperative refraction of the Barrett Universal 2 formula on the biometry printout. In cases of keratometric astigmatism exceeding 0.75 diopters (D), the toric version of the respective IOL model was implanted. Established patient selection criteria at the study location to qualify for presbyopia correcting IOL implantation were an expected postoperative corrected distance visual acuity (CDVA) ≥ 0.2logMAR, while exclusion criteria were reduced expected postoperative visual acuity, strabismus, a history of ophthalmic surgery of any kind, and ocular disease other than cataract. In addition, cases with elevated higher-order aberrations (root mean square HOA > 0.4 µm at 4 mm pupil diameter), excessive positive or negative spherical aberration (>±0.5 µm at 6 mm pupil diameter), or patients whose photopic pupil size was outside the range of 2.8 to 6 mm were excluded. All patients had preoperative biometry [IOLMaster 700 (Carl Zeiss Meditec AG, Jena, Germany, Software Version 1.70.16.55256.C70807–1.90.12.5.C87915)], and tomography [Pentacam (Oculus, Wetzlar, Germany), software version 1.31r01], and underwent cataract surgery by phacoemulsification through superior clear corneal incisions.

Quality control measures in our clinic for all new IOL combinations include, as a standard of care, subjective manifest refraction and visual acuity (VA) testing performed by an experienced optometrist or clinician 1, 3, and 6 months after surgery at a lane distance of 4 m, 80 cm, and 40 cm using ETDRS optotype charts designed for the three respective distances. Defocus in the form of the spherical equivalent (SEQ) and blur in the form of the defocus equivalent (DEQ) values were calculated using the manifest refraction results. For each eye, the prediction error (PE) was determined by subtracting the actual postoperative SEQ from the predicted SEQ. For the predicted SEQ, biometry data were retrospectively entered manually into the ESCRS calculator (https://iolcalculator.escrs.org/ (accessed on 30 August 2025–10 September 2025)) and the IOLCON lens power calculator website (https://iolcon.org/lpcm.php (accessed on 30 August 2025–10 September 2025)), using the default constants for each respective IOL.

Photopic monocular and binocular CDVA, distance corrected intermediate visual acuity (DCIVA), and distance corrected near visual acuity (DCNVA) were recorded. Photopic uncorrected distance, intermediate, and near visual acuity (UDVA, UIVA, UNVA) were acquired in the same manner. The patient was then asked to choose the intermediate and near distance with the best VA. These distances were measured, and VA testing was repeated at these distances. One month postoperatively, photopic distance-corrected binocular reading speed was ascertained using the IREST test 6, and contrast sensitivity testing was performed under photopic conditions using a bilateral Pelli–Robson Test. Photopic unilateral and bilateral distance-corrected defocus curves from +1.0 to −4.0 D were performed 6 months after surgery in 0.50 D increments.

Data were analyzed using Microsoft Excel and SPSS software (SPSS V 24.0; IBM, Armonk, New York, NY, USA). Descriptive statistics are provided via tables. The Shapiro–Wilk test was used to assess the normality of the distribution of metric variables. Visual acuity at various defocus steps and at multiple distances was compared using either a Wilcoxon signed rank (paired) or a Wilcoxon rank sum (unpaired) test. To assess the statistical significance of differences in partly bilateral data for prediction errors, we performed generalized linear mixed models with subject ID as a random effect. This models within-subject correlation between eyes while including unilateral cases. Post hoc pairwise comparisons were conducted on subject-level aggregated data using the Wilcoxon signed-rank test. The significance level for this study was Qt at *p* < 0.05. Multiple testing correction was applied using the Benjamini-Hochberg procedure.

## 3. Results

### 3.1. Demographics

In total, 115 patients met the criteria for inclusion in the study. Fifty-six and 59 patients received bilateral LISA TRI or Comfort IOLs, respectively. There were no intra- or postoperative complications. Table 1 summarizes the demographic and preoperative biometric parameters for each group. Supplementary Table S1 summarizes the quality of biometry measurements used for IOL calculations.

### 3.2. Results with New Generation IOL Formulas

The accuracy of IOL calculation with either the ESCRS calculator or the IOLcon LPCM can be deduced from Table 2. Formulas are ranked by mean absolute error (MAE). Significant offsets were observed in 3 formulas (K6, Castrop, Hoffer QST) for the LISA TRI and in all five formulas for the Comfort. The absolute prediction error was significantly lower for all formulas for the LISA TRI compared to all formulas for the Comfort, except the K6 formula.

### 3.3. Refractive Outcomes: SEQ, Astigmatism, DEQ

Mean values of SEQ, astigmatism magnitude and DEQ diverging from a plano refraction are displayed in Supplementary Table S2. The proportion of eyes within an absolute prediction error of 0.25 D, 0.50 D, 0.75 D and 1.0 D at selected postoperative time points are displayed in Table 3. The stability of the postoperative refraction is displayed in Table 4.

### 3.4. Defocus Curves

**Figure 1:**
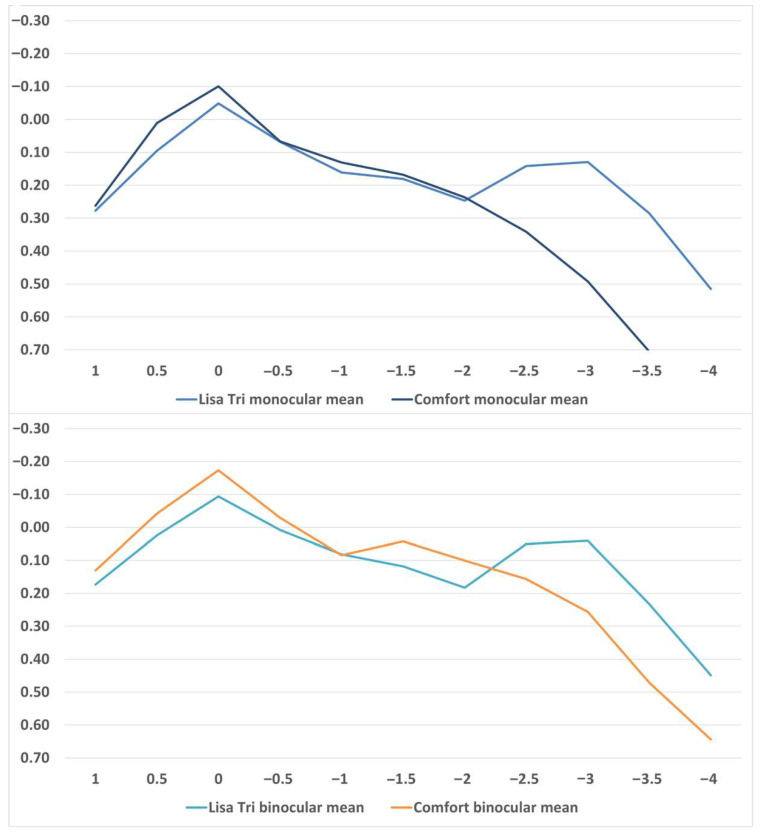
Defocus curves of binocular and monocular LISA TRI and comfort implantation.

The monocular and binocular mean defocus curves for both IOLs are displayed in Figure 1. Numerical values can be found in Table 5. There were no significant differences for +1.0 D and +0.5 D, and for −0.5 D, −1.0 D, −1.5 D, and −2.0 D. There was a significant difference for 0 D, −2.5 D, −3.0 D, −3.50 D, and −4.0 D.

### 3.5. Near, Intermediate and Distance Visual Acuity

Distance corrected and uncorrected near, intermediate and distance visual acuity are displayed in Supplementary Table S3. Preferred distances for near and intermediate visual acuity are reported in Supplementary Table S4. No statistically significant differences were found in CDVA and DCIVA between either group, but a statistically significant difference was observed in DCNVA.

### 3.6. Photopic Reading Speed and Contrast Sensitivity

LISA TRI: At 60 cm, the binocular distance-corrected mean photopic reading speed was 125.01 ± 51.39 words/minute. In binocular photopic contrast sensitivity testing, patients recognized an average of 33.75 ± 2.66 optotypes (min 30; max 36) correspond to a mean logarithmic contrast sensitivity of 1.69 log units (min 1.50; max 1.80). Statistically significant differences were observed only in reading speed at 60 cm between both groups.

Comfort: At 60 cm, the binocular distance-corrected mean photopic reading speed was 145.31 ± 27.19 words/minute. In binocular photopic contrast sensitivity testing, patients recognized an average of 35.75 ± 3.24 optotypes (min 30; max 36) corresponding to a mean logarithmic contrast sensitivity of 1.79 log units (min 1.50; max 1.80). No statistically significant differences were found in contrast sensitivity between the groups.

## 4. Discussion

This study compared real-world refractive and visual outcomes between the trifocal diffractive AT LISA TRI and the nondiffractive EDOF Comfort IOL. The major findings were that the LISA TRI demonstrated higher predictive accuracy and faster refractive stabilization. Furthermore, the Comfort EDOF IOL showed greater variability in early postoperative refraction, and defocus curve analysis confirmed the expected optical performance differences, with superior near visual acuity for the LISA TRI and better distance visual acuity for the Comfort IOL.

The evaluation of modern IOL formula accuracy has become a central topic in ophthalmic literature. To date, there is no standardized recommendation regarding the choice of formula. A recent systematic review, which included papers from 2015 to 2022, concluded that axial length and corneal curvature should be key considerations [16]. Achieving accurate postoperative refractive stability is essential for the success of mIOLs and EDOF-IOLs. To minimize the deviation of the predicted refraction from the postoperative refraction and thus ultimately the satisfaction of the patients concerned, IOL formulas are constantly being refined and optimized [17]. Besides several biometric parameters, formula accuracy may also vary with the specific IOL platform used and the IOL power distribution of the study sample [17].

Our data show high predictive accuracy for modern IOL power calculation formulas in both IOL groups. The LISA TRI IOL demonstrated noticeably high refractive predictability (Table 2). In search of an explanation, we were unable to find a single reason. PEs for this IOL were consistently low, and the MAE and SD confirm a high level of precision. On one hand, this could be attributable to the excellent biometric quality of our mIOL study population, with high VA, regular corneal shapes and reliable measurements [18]. Refractions were conducted by an independent, non-surgical optometrist, excluding bias from lens selection or surgical planning. We initially considered whether the subjective refraction method or a tendency toward fogging with minus lenses (instead of fogging with plus glasses) might have influenced the outcomes, particularly in the multifocal IOL group. However, the defocus curve analysis does not support this assumption: the curves were as expected, without indications of systematic defocus errors. Still, as a retrospective group, there might have been a tendency to subconsciously refract the patient towards 0 D, to enhance patient satisfaction with their mIOL result. On the other hand, this does not seem likely as the same bias could not be found in the Comfort. Speaking for the highly accurate outcomes, only 2 patients required postoperative enhancement via excimer laser vision correction.

Notably, some systematic offsets were observed, especially with certain formulas in specific IOL groups, which suggests possible inadequacies in the lens constants used (Table 2). For example, the Castrop and K6 formulas showed low SDs and high theoretical accuracy for the LISA TRI (lower SD than Barrett), yet small but consistent mean errors point toward the need for optimized constants. The Hoffer QST and PEARL-DGS showed slightly higher SD, but only the Hoffer QST had an offset in mean PE. For the Comfort IOL, all formulas but Barrett showed an offset with a mean PE > 0.15 D. IOL constants for these formulas could be optimized to achieve better results. All formulas for the Comfort IOL would benefit from optimized constants in our cohort to achieve even better results.

A marked difference in postoperative refractive stability was noted between the two IOLs. The LISA TRI group exhibited early refractive changes exceeding 0.5 D in about 20% of cases at one month, but these fluctuations largely resolved by the three-month follow-up. In contrast, the Comfort IOL showed persistent refractive instability, with approximately 13% of eyes still experiencing SEQ changes >0.5 D even at three months and 23% after three months (Table 4). These findings carry important clinical implications: optical adaptations and enhancements should be timed cautiously—delayed in the case of the LISA TRI until refractive stability is achieved and potentially anticipated for the Comfort IOL due to its ongoing variability. On the other hand, a possible explanation for the greater variability with the Comfort group is that the refractive endpoint is slightly more ambiguous with an EDOF lens than with a multifocal lens, which has very distinct focal points.

While the SEQ is commonly reported in studies, we emphasize that the DEQ has been shown to be a more clinically relevant parameter, especially in refractive procedures, showing a higher correlation of the postoperative UDVA to the DEQ than the SEQ [19]. The DEQ is a metric that captures overall optical blur and offers a more balanced assessment of refractive error, especially in patients with multifocal or EDOF IOLs. DEQ is derived from the power vector components of spectacle refraction and provides a better representation of a patient’s visual performance than SEQ alone. In our data, DEQ showed consistently fewer eyes within 0.5 D than SEQ and higher absolute values than SEQ, underscoring the importance of integrating DEQ in accuracy reporting (Supplementary Tables S2 and S3). Relying solely on SEQ could be misleading in overestimating the success rate of presbyopia-correcting IOL implantation. Our data further confirm that residual astigmatism is less predictable than SEQ, a trend in line with prior literature (Table 3). The upper limits observed for postoperative cylinder (approximately 60% within 0.5 D) align with those reported in large-scale studies, confirming the robustness of our surgical and planning protocols [20,21]. Astigmatism prediction remains more challenging than SEQ due to its susceptibility to numerous variables, including preoperative keratometry, rotational stability of toric IOLs, internal astigmatism and individual wound healing response [19,22,23,24,25,26]. Furthermore, surgically induced astigmatism affects both the magnitude and the axis of the postoperative astigmatism, with postoperative axis >10° occurring in over 30% of patients, as demonstrated in prior studies [24,27].

Our defocus curve findings for the LISA TRI and Comfort IOLs were consistent with earlier publications, reinforcing the reproducibility of their optical profiles [3,4,5,19,20,21,28,29,30]. As expected, the defocus curve analysis reflected the optical design differences between the lenses: the EDOF IOL provided slightly better distance visual acuity, whereas the mIOL demonstrated superior intermediate and near performance, with a distinct peak at approximately −2.5 D defocus, indicating a broader depth of field (Figure 1 and Table 5). Reading speed for Lisa Tri was comparable to other studies [31].

It’s pivotal to note that the optical design of the Comfort IOL differs fundamentally from that of diffractive EDOF IOLs. The Comfort employs a refractive, sectorial, rotationally asymmetric design with a +1.5 D near addition to extend depth of focus without diffractive rings, which may induce lower photic phenomena but reduced near visual acuity. In contrast, diffractive EDOF IOLs use echelette or ring-based structures to elongate the focal area, often resulting in better near vision at the cost of more halos or glare. Other non-diffractive EDOF IOL designs, such as those based on higher-order aberration modulation, follow different optical principles but share the goal of minimizing photic phenomena while improving intermediate vision [32,33].

The main limitation of this study is its retrospective, single-center design, with all surgeries performed by a single experienced surgeon. This may limit generalizability and introduce a potential operator-related bias, as surgical technique, experience, and patient counseling could have influenced postoperative outcomes. Future multicenter and multi-surgeon prospective studies are warranted to validate these findings under broader clinical conditions. Potential selection bias should be acknowledged, as patients with a strong preference to avoid possible dysphotopsias were less likely to receive the multifocal IOL. Additionally, the PEARL-DGS formula in the ESCRS calculator was updated at the time of this study, but the previous formula was still used in this study. A key strength of this study is the inclusion of a large cohort and the evaluation of DEQ alongside SEQ, providing a more nuanced understanding of refractive outcomes. DEQ showed a lower proportion of eyes within 0.5 D than SEQ. Our results showed that the LISA TRI appears to have relative refractive stability after 3 months. After 1 month, a change of 0.5 D SEQ was still present in approximately 14% of patients. In contrast to the Comfort, SEQ fluctuations were still present in 13% of patients after 3 months. These findings are clinically relevant and should be considered in the post-operative procedure with dissatisfied patients.

## Figures and Tables

**Table 1:** Demographic and preoperative biometric parameters. Values are presented as mean ± standard deviation unless otherwise noted.

	Parameter	LISA TRI (*n* = 56)	Comfort (*n* = 59)
Demographics	Sex (Female/Male)	42.8%/57.2%	47.9%/52.1%
	Age at Surgery (years)	61.25 ± 10.17	66.73 ± 9.83
IOL Data	IOL Power (D)	20.77 ± 3.11	19.26 ± 4.44
Scheimpflug Tomography Data	Pupil Size (mm)	3.80 ± 1.58	3.76 ± 1.77
	SA (6 mm)	0.33 ± 0.12	0.38 ± 0.15
	RMS HOA (4 mm)	0.19 ± 0.07	0.21 ± 0.08
Ocular Biometry	AL (mm)	23.71 ± 1.16	23.81 ± 1.57
	K_mean_ (D)	43.62 ± 1.39	43.80 ± 1.70
	ACD (mm)	3.32 ± 0.37	3.10 ± 0.43
	LT (mm)	4.28 ± 0.34	4.61 ± 0.43
	CCT (mm)	0.54 ± 0.04	0.55 ± 0.03
	WTW (mm)	12.13 ± 0.41	12.02 ± 0.43

D = diopters; IOL = intraocular lens; SA = spherical aberration; RMS HOA = root-mean-squared higher order aberrations; AL = axial length; K_mean_ = mean keratometry; ACD = anterior chamber depth; LT = lens thickness; CCT = central corneal thickness; WTW = white-to-white.

**Table 2:** Accuracy of IOL calculation for both IOL types. Formulas were entered into the ESCRS online calculator and the IOLcon online lens power calculation module. The default IOL constants were used. There were no default constants for the Castrop formula for the Comfort IOL. Hence, no calculations were carried out.

	MAE	RMSE	PE-ME	PE-SD	PE-Median	PE-IQR	PE 95%CI UB	PE 95%CI LB	Median AE	AE 95%CI UB	AE 95%CI LB	%AE ≤ 0.25 D	%AE ≤ 0.50 D	%AE ≤ 0.75 D	%AE ≤ 1.00 D
LISA TRI 839 MP IOL															
EVO 2.0	0.254	0.345	0.015	0.350	0.045	0.145	0.526	−0.521	0.190	0.742	0.014	57.143	91.43	97.14	97.14
Kane	0.264	0.341	0.027	0.345	0.090	0.150	0.499	−0.555	0.215	0.666	0.026	57.14	94.29	97.14	97.14
Barrett	0.270	0.361	0.023	0.365	0.075	0.115	0.603	−0.525	0.200	0.746	0.014	60.00	88.57	97.14	97.14
K6	0.281	0.360	0.133	0.340	0.145	0.165	0.640	−0.491	0.230	0.763	0.024	51.43	80.00	97.14	100
Castrop	0.297	0.406	0.208	0.354	0.162	0.319	0.812	−0.371	0.209	0.927	0.004	57.14	74.29	88.57	100
PEARL-DGS	0.303	0.397	0.070	0.397	0.090	0.208	0.738	−0.572	0.250	0.929	0.005	54.29	91.43	94.29	97.14
Hoffer QST	0.351	0.442	0.178	0.411	0.22	0.133	0.926	−0.546	0.295	1.068	0.042	40.00	82.86	88.57	94.29
Comfort (LS 313-MF15) IOL															
K6	0.317	0.410	0.164	0.381	0.170	0.160	0.883	−0.515	0.265	0.883	0.010	48.72	84.62	87.18	97.44
Barrett	0.327	0.410	0.139	0.391	0.115	0.300	0.878	−0.622	0.240	0.878	0.043	51.28	76.92	92.31	97.44
Kane	0.333	0.429	0.240	0.360	0.180	0.230	0.901	−0.412	0.270	0.901	0.035	48.72	82.05	87.18	97.44
EVO 2.0	0.341	0.442	0.251	0.369	0.205	0.205	1.138	−0.409	0.285	1.138	0.010	48.72	82.05	87.18	94.87
PEARL-DGS	0.369	0.485	0.285	0.398	0.260	0.203	1.197	−0.462	0.285	1.197	0.010	43.59	76.92	84.62	92.31

MAE = mean absolute error; RMSE = root mean squared error; PE = prediction error; ME = mean; SD = standard deviation; IQR = interquartile range; CI = confidence interval; UB = upper bound; LB = lower bound; AE = absolute error.

**Table 3:** Percentages of eyes within absolute residual refraction ranges for SEQ, CYL, and DEQ at 1 month, 3 months, and 6 months postoperatively.

		LISA TRI	Comfort IOL
SEQ (1 m/3 m/6 m)	%±0.25 D	38/55/48	46/49/59
	%±0.5 D	67/82/80	70/75/76
	%±0.75 D	84/94/93	85/86/86
	%±1.0 D	93/96/98	87/90/94
CYL (1 m/3 m/6 m)	%±0.25 D	27/37/48	33/41/24
	%±0.5 D	60/59/69	55/61/43
	%±0.75 D	78/84/83	70/81/63
	%±1.0 D	89/92/94	84/93/82
DEQ (1 m/3 m/6 m)	%±0.25 D	16/22/24	23/29/27
	%±0.5 D	42/59/50	47/58/51
	%±0.75 D	67/76/85	63/71/67
	%±1.0 D	85/96/98	78/81/80

D = diopters, SEQ = spherical equivalent, CYL = refractive astigmatism magnitude, DEQ = defocus equivalent; m = month.

**Table 4:** Percentage of eyes with changes in SEQ and refractive astigmatism magnitude (CYL) within error thresholds between time points of 1 month, 3 months, and 6 months.

		LISA TRI IOL			Comfort IOL		
		1 v 3	1 v 6	3 v 6	1 v 3	1 v 6	3 v 6
SEQ	%±0.25 D	69	71	81	63	59	69
	%±0.5 D	86	84	100	87	81	77
	%±0.75 D	94	94	100	97	96	96
	%±1.0 D	100	97	100	100	100	96
CYL	%±0.25 D	74	71	77	67	70	73
	%±0.5 D	97	94	90	93	85	88
	%±0.75 D	97	100	100	100	100	96
	%±1.0 D	100	100	100	100	100	100

D = diopters, SEQ = spherical equivalent, CYL = refractive astigmatism magnitude, v = versus.

**Table 5:** Numerical values of monocular and binocular defocus curves with a defocus of +1.0 D to −4.0 D in 0.5 D increments. Visual acuity is displayed in logMAR. Values are presented as the mean, standard deviation (SD), median, and interquartile range (IQR).

Defocus (D)	+1.0	+0.5	0	−0.5	−1	−1.5	−2	−2.5	−3	−3.5	−4
AT LISA TRI Binocular											
Mean	0.17	0.02	−0.09	0.01	0.08	0.12	0.18	0.05	0.04	0.23	0.45
SD	0.12	0.06	0.05	0.05	0.06	0.09	0.11	0.07	0.06	0.11	0.10
Median	0.10	0.00	−0.10	0.00	0.10	0.10	0.20	0.00	0.05	0.20	0.40
IQR	0.20	0.10	0.00	0.00	0.02	0.13	0.13	0.10	0.10	0.13	0.12
Comfort Binocular											
Mean	0.13	−0.04	−0.17	−0.03	0.08	0.04	0.10	0.16	0.26	0.47	0.64
SD	0.13	0.05	0.05	0.08	0.07	0.10	0.14	0.10	0.11	0.16	0.13
Median	0.10	0.00	−0.20	0.00	0.10	0.00	0.10	0.20	0.20	0.40	0.60
IQR	0.11	0.10	0.05	0.00	0.05	0.10	0.20	0.10	0.15	0.25	0.05
LISA TRI Monocular											
Mean	0.29	0.10	−0.05	0.07	0.17	0.19	0.26	0.15	0.13	0.30	0.54
SD	0.13	0.12	0.05	0.07	0.09	0.10	0.11	0.13	0.14	0.14	0.13
Median	0.30	0.10	−0.08	0.10	0.10	0.20	0.30	0.10	0.10	0.30	0.49
IQR	0.20	0.10	0.10	0.10	0.12	0.20	0.10	0.10	0.10	0.20	0.20
Comfort Monocular											
Mean	0.26	0.01	−0.10	0.07	0.13	0.17	0.24	0.34	0.49	0.71	0.76
SD	0.19	0.09	0.05	0.07	0.12	0.16	0.17	0.20	0.18	0.22	0.21
Median	0.30	0.00	−0.10	0.10	0.10	0.20	0.20	0.30	0.49	0.70	0.70
IQR	0.15	0.10	0.01	0.10	0.15	0.20	0.20	0.25	0.15	0.35	0.20

## Data Availability

The data that support the findings of this study are available from the corresponding author upon reasonable request.

## Supplementary Materials

The following supporting information can be downloaded at: https://www.mdpi.com/article/10.3390/jcm14228259/s1, Table S1. Percentage of successful biometric measurements and system warnings by device parameter; Table S2. Postoperative subjective refraction outcomes for spherical equivalent (SEQ), astigmatism magnitude (CYL), and defocus equivalent (DEQ) at 1, 3, and 6 months; Table S3. Uncorrected and distance-corrected Visual acuity (logMAR) for three distances; Table S4. Preferred uncorrected and distance-corrected viewing distances (in cm).
