# Supplementary material for: Real-World Visual and Refractive Results of Two Different Presbyopia Correcting Intraocular Lenses
Source: J Clin Med. 2025 Nov 20;14(22):8259. doi: 10.3390/jcm14228259 (PMC12653068; doi:10.3390/jcm14228259)
Supplement: Supplementary file 1 [file jcm-14-08259-s001.zip › jcm-3950473-supplementary.pdf]

	AL		ACD		LT		R		CCT		WTW	
	Successful (%)	Warning (%)	Successful (%)	Warning (%)	Successful (%)	Warning (%)	Successful (%)	Warning (%)	Successful (%)	Warning (%)	Successful (%)	Warning (%)
**LISA TRI Overall**	98.31	1.69	95.76	4.24	92.37	7.63	91.53	8.47	98.31	1.69	99.15	0.85
**LISA TRI stigmatic**	97.56	2.44	95.12	4.88	91.46	8.54	91.46	8.54	97.56	2.44	100	0
**LISA TRI astigmatic**	100	0	97.22	2.78	94.44	5.56	91.67	8.33	100	0	97.14	2.86
**Comfort overall**	96.50	3.50	97.20	2.80	86.71	13.29	94.41	5.59	99.30	0.70	96.50	3.50
**Comfort stigmatic**	95.83	4.17	100	0	81.25	18.75	95.83	4.17	100	0	95.83	4.17
**Comfort astigmatic**	96.84	3.16	95.79	4.21	89.47	10.53	93.68	6.32	98.95	1.05	96.84	3.16

Supplementary Table S1. Percentage of successful biometric measurements and system warnings by device parameter.

AL = axial length, ACD = anterior chamber depth, LT = lens thickness, R = radius of corneal curvature , CCT = central cornea thickness, WTW = white-to-white

	**LISA TRI IOL**<br><br>**(Mean ± SD)**	**Comfort IOL**<br><br>**(Mean ± SD)**
**SEQ 1 months postOP (D)**	-0.33 ± 0.52	-0.38 ± 0.72
**SEQ 3 months postOP (D)**	-0.18 ± 0.45	-0.03 ± 0.62
**SEQ 6 months postOP (D)**	-0.22 ± 0.41	-0.07 ± 0.50
**CYL 1 months postOP (D)**	0.57 ± 0.41	0.64 ± 0.49
**CYL 3 months postOP (D)**	0.55 ± 0.41	0.54 ± 0.42
**CYL 6 months postOP (D)**	0.49 ± 0.34	0.73 ± 0.46
**DEQ 1 months postOP (D)**	0.76±0.46	0.86 ± 0.71
**DEQ 3 months postOP (D)**	0.63±0.38	0.71 ± 0.50
**DEQ 6 months postOP (D)**	0.62±0.32	0.71 ± 0.46

Supplementary Table S2. Postoperative subjective refraction outcomes for spherical equivalent (SEQ), astigmatism magnitude (CYL), and defocus equivalent (DEQ) at 1, 3, and 6 months.

D = diopters; EDOF = extended depth-of-focus; IOL = intraocular lens; SD = standard deviation; SEQ = spherical equivalent; DEQ = defocus equivalent

		UDVA	UIVA	UNVA	CDVA	DCIVA	DCNVA
LISA TRI	3m	0.12±0.14	0.14±0.14	0.15±0.13	-0.01±0.08	0.07±0.11	0.06±0.16
	6m	0.10±0.14	0.11±0.10	0.16±0.12	-0.03±0.06	0.08±0.10	0.03±0.08
Comfort	3m	0.15±0.20	0.04±0.07	0.40±0.23	0.00±0.09	0.07±0.10	0.29±0.25
	6m	0.12±0.20	0.12±0.35	0.25±0.14	-0.03±0.09	0.14±0.40	0.29±0.12

Supplementary Table S3. Uncorrected and distance-corrected Visual acuity (logMAR) for three distances.

Distance Type	Time Point	LISA TRI (Mean ± SD)	Comfort (Mean ± SD)
UIVA	3 months	69.20 ± 5.53	73.61 ± 6.71
	6 months	74.92 ± 6.76	73.64 ± 5.64
UNVA	3 months	36.56 ± 3.18	38.45 ± 3.21
	6 months	33.67 ± 4.01	39.64 ± 1.21
DCIVA	3 months	70.13 ± 5.54	70.35 ± 6.32
	6 months	72.22 ± 6.09	70.95 ± 5.94
DCNVA	3 months	37.76 ± 2.77	45.64 ± 16.76
	6 months	36.44 ± 3.36	39 ± 3.00

Supplementary Table S4. Preferred uncorrected and distance-corrected viewing distances (in cm).
